# Metabolomic profiling of CSF and blood serum elucidates general and sex-specific patterns for mild cognitive impairment and Alzheimer’s disease patients

**DOI:** 10.3389/fnagi.2023.1219718

**Published:** 2023-08-24

**Authors:** Georgy Berezhnoy, Christoph Laske, Christoph Trautwein

**Affiliations:** ^1^Werner Siemens Imaging Center, Department of Preclinical Imaging and Radiopharmacy, University Hospital Tübingen, Tübingen, Germany; ^2^Section for Dementia Research, Hertie Institute for Clinical Brain Research and Department of Psychiatry and Psychotherapy, University Hospital Tübingen, Tübingen, Germany; ^3^German Center for Neurodegenerative Diseases (DZNE), Tübingen, Germany

**Keywords:** brain, dementia, cerebrospinal fluid, metabolism, neurodegeneration, memory, theranostics

## Abstract

**Background:**

Beta-amyloid (Abeta) and tau protein in cerebrospinal fluid (CSF) are established diagnostic biomarkers for Alzheimer’s disease (AD). However, these biomarkers may not the only ones existing parameters that reflect Alzheimer’s disease neuropathological change. The use of quantitative metabolomics approach could provide novel insights into dementia progression and identify key metabolic alterations in CSF and serum.

**Methods:**

In the present study, we quantified a set of 45 metabolites in CSF (71 patients) and 27 in serum (76 patients) in patients with mild cognitive impairment (MCI), AD, and controls using nuclear magnetic resonance (NMR)-based metabolomics.

**Results:**

We found significantly reduced CSF (1.32-fold, *p* = 0.0195) and serum (1.47-fold, *p* = 0.0484) levels of the ketone body acetoacetate in AD and MCI patients. Additionally, we found decreased levels (1.20-fold, *p* = 0.0438) of the branched-chain amino acid (BCAA) valine in the CSF of AD patients with increased valine degradation pathway metabolites (such as 3-hydroxyisobutyrate and α-ketoisovalerate). Moreover, we discovered that CSF 2-hydroxybutyrate is dramatically reduced in the MCI patient group (1.23-fold, *p* = 0.039). On the other hand, vitamin C (ascorbate) was significantly raised in CSF of these patients (*p* = 0.008). We also identified altered CSF protein content, 1,5-anhydrosorbitol and fructose as further metabolic shifts distinguishing AD from MCI. Significantly decreased serum levels of the amino acid ornithine were seen in the AD dementia group when compared to healthy controls (1.36-fold, *p* = 0.011). When investigating the effect of sex, we found for AD males the sign of decreased 2-hydroxybutyrate and acetoacetate in CSF while for AD females increased serum creatinine was identified.

**Conclusion:**

Quantitative NMR metabolomics of CSF and serum was able to efficiently identify metabolic changes associated with dementia groups of MCI and AD patients. Further, we showed strong correlations between these changes and well-established metabolomic and clinical indicators like Abeta.

## Graphical Abstract

MCI and AD patients’ health and disease progression majorly depends on genetic predisposition (ApoE4) and individual lifestyle factors (metabolites, oxidative stress, amyloid plaque formation, and tau protein build-up). NMR-based metabolomics combined with standardization in sample preparation and analysis allows to phenotype metabolic shifts in CSF and blood. In CSF we monitored a decrease in acetoacetate, a ketone body, in both MCI and AD. We furthermore identified low 2-hydroxybutyrate, which can mark insulin resistance, in MCI. Finally, 1,5-anhydrosorbitol, a metabolite from the polyol pathway is higher exclusively in dementia (AD & MCI) males. In blood serum, we identified lower acetoacetate in the MCI group, lower blood serum ornithine in the AD group, higher glucose in MCI, and an increase of creatine in AD females when compared to AD males. In conclusion, we added a scheme of metabolomic transitions to highlight the close position of valine and valine degradation products to various key metabolites (Krebs cycle metabolites, ketone bodies, glucose, pyruvate, acetyl-CoA, and glutamic acid). 
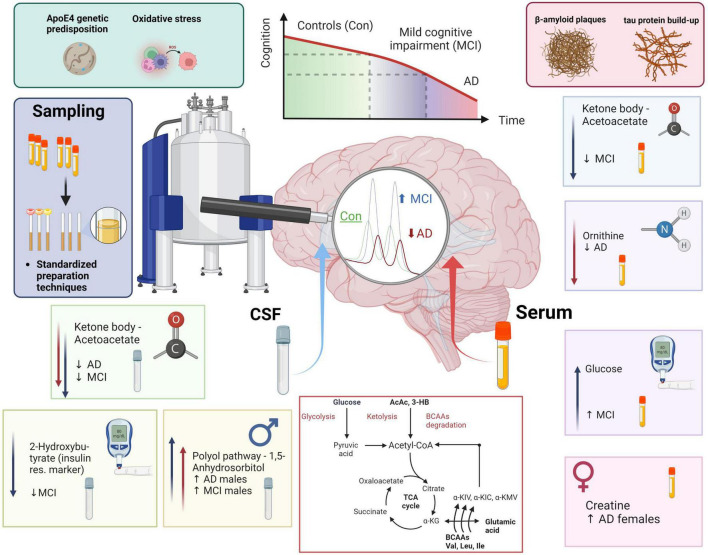

## Introduction

Alzheimer’s disease (AD) is the most common cause of dementia globally (Apostolova, 2016). AD is characterized by a substantial loss of neurons and synapses followed by severe brain atrophy in later stages (Serrano-Pozo et al., 2011). Beta-amyloid (Abeta) and tau proteins in cerebrospinal fluid (CSF) are established diagnostic biomarkers for AD (Mattsson, 2009). However, applying new omics approaches like metabolomics can provide a much broader readout of vital brain tissue functions (Bagyinszky et al., 2020; Higginbotham et al., 2020; Clark et al., 2021; Libiger et al., 2021). A reliable quantitative key metabolism parameter set could decipher neuro-damaging brain metabolism (e.g., mitochondrial function decline, oxidative stress, nucleic and amino acid metabolism disruption). In animal models of AD, there were monitored alterations of energetic metabolic molecules through the lens of brain tissue metabolomics (Trushina et al., 2012). In the human population, a novel approach for AD has an uplifting potential to be utilized as the cutting-edge part of clinical diagnostics. Of note, the addition of metabolomic markers based on the metabolomics screening of the same patient cohort, for which a set of AD biomarkers had been identified, was yet increasing the overall discriminatory model of AD, mild cognitive impairment (MCI), and controls (Jääskeläinen et al., 2020). Thus, CSF metabolomic analysis in AD may provide a more comprehensive understanding of cognitive impairment in a combination with blood metabolomics, in the future, also allowing to perform a patient-specific inquiry.

A wide range of analytical techniques and matrices (e.g., biofluids or tissue) can be used to generate a metabolomics knowledge base (Evans et al., 2020). Hereby, the use of nuclear magnetic resonance (NMR) metabolomics, compared to mass spectrometry, provides non-destructive sample preparation, batch reproducibility, and the possibility of straightforward metabolite identification and quantification (French et al., 2018). NMR-based metabolomics has been used for AD research (Jääskeläinen et al., 2020; Vignoli et al., 2020) and diagnostics of disease progression (Sinclair et al., 2009; Kork et al., 2012). In-depth meta-analysis can elucidate individual patients’ phenotypes and develop targets for personalized precision medicine approaches. Previous NMR studies performed in CSF have shown several affected metabolic pathways altered in both AD and MCI. The main findings include impairment of glucose metabolism (Mullins et al., 2018), arginine-tryptophan metabolism (Trushina et al., 2013), and acetate metabolism (Vignoli et al., 2020).

Several publications distinguish MCI and AD groups by CSF metabolic fingerprints (Trushina et al., 2013; Jääskeläinen et al., 2020; Vignoli et al., 2020). Additionally, recent research has focused on developing sensitive and specific metabolic levels in blood samples for diagnostics of AD and MCI (Proitsi et al., 2017; Weng et al., 2019; Yilmaz et al., 2020; Blasko et al., 2021; Peña-Bautista et al., 2021). One study identified a significant correlation between branched-chain amino acids (BCAAs) and the risk of dementia progression (Tynkkynen et al., 2018). Furthermore it has been found that blood lipoprotein alterations are hallmarks for also sex-based features of mild cognitive impairment and AD (Berezhnoy et al., 2022).

In this study, we applied quantitative NMR-based metabolomics of cerebrospinal fluid and serum to elucidate novel dementia-specific alterations in metabolism and correlated those with well-established clinical parameters such as Abeta and tau. Of note, this study is based on an *in vitro* diagnostics research (IVDr) standard operation procedure (SOP) of CSF and serum aliquots’ sample preparation technique with further profiling of NMR spectra. This standardized approach guarantees the highest reliability and reproducibility (Albrecht et al., 2020). Additionally, using an IVDr and SOP-derived data set can help to construct a knowledge base for NMR metabolomics in body fluids discriminating various diseases.

## Materials and methods

Three clinical groups were investigated within this study: control (Con) subjects, MCI patients, and AD patients.

This study was performed in accordance with the Declaration of Helsinki. All participants gave their written consent and samples were collected by the biofluid biobank at the Hertie Institute for Clinical Brain Research, the Center of Neurology, University of Tübingen, Tübingen, Germany. In total, 71 CSF and 76 serum samples were investigated. The participants were divided into different sub-cohorts as illustrated in Table 1 according to a metadata panel with clinical CSF biomarkers for AD-driven dementia consisting of Abeta1–42 (>800 pg/mL threshold), h-Tau (<300 pg/mL threshold, total human tau protein amount), and p-Tau (<60 pg/mL threshold, phosphorylated tau protein amount). The following additional metadata parameters were received and considered for analysis: age, sex status (0 – male, 1 – female, for the purpose of categorization within statistical software), amyloid positivity status (AmPos), mini-mental state examination (MMSE) score, cholinesterase inhibitor drug treatment positivity status (chol-est inh), number of ApoE4 alleles, hypercholesterolemia and statin drug treatment status. AmPos was operated as a categorical value in contrast to the continuous row of Abeta1–42 values and was generated based on operated threshold (<650 pg/ml) for defying amyloid positivity in patients, as has been published before (Düzel et al., 2022). For clinical needs, this difference could make a difference, and therefore both amyloid (Abeta) values and patients’ statuses were given in the current work.

**TABLE 1:** Clinical data of the CSF and blood serum study cohort for three studied groups –AD and MCI diagnosed patients, and Con samples from hospital patients with various comorbidities that are not affecting poor CSF biomarker scores.

	Total	Con	MCI	AD
**CSF cohort**				
Patients	71	20	22	29
Male	43	13	16	14
Female	28	7	6	15
Age (mean ± SD)	68.0 ± 8.2	69.7 ± 7.0	69.5 ± 9.8	65.6 ± 7.3*
MMSE (mean ± SD)			27.0 ± 1.9	22.5 ± 3.6 ***
**Blood serum cohort**				
Patients	76	29	21	26
Male	38	15	15	13
Female	33	14	6	13
Age (mean ± SD)	69.0 ± 7.7	71.7 ± 4.6	69.3 ± 10.0	65.9 ± 7.5***
MMSE (mean ± SD)	26.4 ± 3.4	29.2 ± 0.9	26.9 ± 1.9***	22.8 ± 3.4***

Herein, the MMSE scores have been added according to the available entries from the patient groups. Statistical significance (in comparisons with the Con group, while CSF MMSE scores were compared between AD and MCI groups): *P ≤ 0.05, ***P ≤ 0.001; SD, standard deviation.

Cerebrospinal fluid and serum aliquots were stored at −80°C and transported on dry ice until preparation for NMR analysis. All samples were prepared according to a commercial SOP for CSF and serum (AVANCE IVDr Methods Version 003, Bruker BioSpin GmbH, Ettlingen, Germany). In brief, on the day of preparation, the samples were thawed at room temperature and then directly prepared. Each CSF sample (200 μL) was mixed with a pH neutral (pH = 7.40) CSF preparation buffer (40 μL, ordering number AH0623-10, provided by Bruker BioSpin GmbH, Ettlingen, Germany), containing 60% deuterium oxide, 0.9 mM potassium monophosphate, 0.06% sodium 3-(trimethylsilyl)-2,2,3,3-tetradeuteropropionate (TSP) and 1.2 mM bacteriostatic sodium azide. Serum samples (350 μL) were mixed with a pH neutral (pH = 7.40) plasma/serum preparation buffer (350 μL, ordering number AH0622-10, provided by Bruker BioSpin GmbH, Ettlingen, Germany), containing 20% deuterium oxide, 0.075M sodium monophosphate, 4.6 mM sodium 3-(trimethylsilyl)-2,2,3,3-tetradeuteropropionate (TSP) and 0.04% bacteriostatic sodium azide, as published previously (Dona et al., 2014).

After thoroughly mixing (no vortexing!), a portion of the resulting mixture (200 μL for the CSF sample, 600 μL for the serum sample) was transferred into a 4″ (3 mm – CSF, 5 mm – serum) NMR glass tube and then placed into an autosampler (Bruker SampleJet™, Bruker BioSpin AG, Fällanden, Switzerland). The Bruker SOP instructs to operate with a higher volume of a sample prepared and then transfer a lesser amount into an NMR tube for better analytical reproducibility and allows to hold an additional technical aliquot. Samples for NMR metabolomic analysis were stored at 6°C in the autosampler prior to analysis and kept for 5 min inside the NMR probe head to reach temperature stability.

Nuclear magnetic resonance experiments were accomplished on a Bruker Avance III HD 600 MHz NMR spectrometer (Bruker BioSpin AG, Fällanden, Switzerland). Samples were measured with a 5 mm TXI probe, using Bruker TopSpin version 3.6.1 (serum dataset) and 3.6.2 (CSF dataset), including additionally required IVDr experiments and software plug-ins as provided by Bruker BioSpin GmbH, Ettlingen, Germany. Quality control (QC) was performed regularly and was accomplished within the Bruker IVDr guidelines of QC testing of the spectroscopic system.

Cerebrospinal fluid spectra were recorded using ^1^H NOESY (nuclear Overhauser effect spectroscopy, number of scans = 32, size of one scan = 98,304 data points) experiment at a temperature of 300.0 K. ^1^H NOESY spectra were utilized to generate a prepared dataset for the subsequent manual metabolite annotation and quantification (Chenomx NMR suite 8.5 software). TSP was used as the chemical shift and the shim reference signal as well as the internal standard calibrator. All spectra were manually matched with database entries from the Chenomx and the HMDB (human metabolome database, available online) libraries containing up to 1,000 compounds fit for 600 MHz NMR spectral dataset. The use of the HMDB library was necessary for those CSF metabolites that were presenting a better spectral fit (citric acid) or unique presence (1,5-anhydrosorbitol) in contrast to the other library.

Serum spectra were recorded using ^1^H NOESY (nuclear Overhauser effect spectroscopy, number of scans – 32, size of one scan – 98,304 data points) experiments at a temperature of 310.0 K. ^1^H NOESY spectra were utilized to generate a prepared dataset for the subsequent automated metabolite annotation and quantification (via the IVDr online server provided by Bruker BioSpin GmbH, Ettlingen, Germany).

The annotation and quantification of the CSF spectra resulted in a total number of 45 metabolites that were identified in all spectra. Additionally, quantifiable protein content was evaluated as background hump area in the ^1^H NOESY spectra, as reported previously (Jukarainen et al., 2008).

The annotation and quantification of serum spectra were provided automatically and server-based by Bruker BioSpin GmbH, Ettlingen, Germany. Herein, a total number of 27 metabolites (via Bruker IVDr Quantification In Plasma/Serum, B.I.Quant-PS™, analysis package) were identified and quantified in all spectra, excluding ethanol, glycerol, and metabolites with less than 1/3 of the total cohort presented concentration values (zeros). Additionally, we calculated ratios of some small molecule metabolites that can provide a comprehensive view of blood metabolomic status in patients: the Fisher’s ratio [branched-chain amino acids (valine, leucine, and isoleucine) to aromatic amino acids (phenylalanine and tyrosine) in the serum; formulated in Soeters and Fischer (1976)], the glutamine-glucose ratio (Gln/Glc) and the lactic acid-to-glucose ratio (Lac/Glc) as reported in Kumar et al. (2020).

Statistical analysis was performed with the MetaboAnalyst 5.0 package (Pang et al., 2021). Even though standardized CSF and serum volumes were used, the data set was additionally normalized by the probabilistic quotient normalization (PQN) routine and logarithmically transformed to account for potential dilution effects and make merged data analysis with Abeta and tau markers feasible (Dieterle et al., 2006). Importantly for CSF sample data, we had to utilize unfiltered CSF aliquots as their spectral data was not proven to show a potential problem with metabolite quantification in regard to internal protein content (e.g., human albumin) located at the spectral baseline. The spectral assignment data yet would have been required to be normalized via PQN in regards to several broadening effects occurring in the chemical shift indicator (TSP) region at 0.00 ppm. Moreover, Chenomx software allowed to further fit in the spectral broadness according to the halfwidth of the TSP signal which would improve the further quality of the fitted profiles.

Data from the CSF biomarkers and metadata were normalized via logarithmic scaling in order to monitor changes of multiple magnitude of metabolic data, biomarkers, and metadata. For each comparison, the following set of parameters was determined: *p*-values [students *t*-test and ANOVA (via Fischer’s LSD least significant difference method) analysis of variance], Pearson correlation coefficients (used in PatternHunter plots), oPLS-DA (orthogonal partial least square discrimination analysis), sPLS-DA (sparse partial least square discrimination analysis), AUC (area under the curve) values from the ROC (receiver operating characteristic) analysis and VIP (variable importance in projection) score of metabolites. For combined NMR data and clinical CSF AD biomarkers, we used logarithmic scaling which results in a normal-like distribution of concentrations with subsequent Pearson’s correlation analysis. In order to produce consistent statistical tests [included the false discovery rate (FDR) rate calculations, where applicable], we applied the following set of thresholds: statistical significance threshold for *p*-values *p* < 0.05 and VIP scores threshold for significant findings VIP > 1.0; AUC values threshold for significant findings AUC > 0.70. Additionally, GraphPad Prism 9.0.1 software was used for unpaired *t*-test boxplot and ANOVA boxplot visualization. Several figures in the current work were created with the BioRender.com online-based service.

## Results

### Cohorts description

A total of 71 CSF and 76 serum patient samples (Table 1) were examined for the NMR and metabolomic analysis. Alongside the samples, group labeling, clinical metadata, and demographical parameters of these two cohorts were provided. Notably, the clinical metadata, such as Abeta1–42 and tau markers (CSF h-Tau and CSF p-Tau), were not available for all patients of the full CSF cohort. Three clinical groups were investigated within this study: Con subjects, MCI patients, and AD-diagnosed individuals. Out of the 71 CSF patient samples obtained, only 58 patient sample entries included AD biomarkers in the CSF. Consequently, the availability of this data serves as a constraining constraint. We consequently had 15 controls (9 males and 6 females), 18 MCI (12 males and 6 females), and 25 AD patients (12 males and 13 females) whose CSF biomarker levels were recorded (Abeta, tau). Measurements of Abeta1–42 (controls – 919 ± 194 pg/mL, MCI – 715 ± 402 pg/mL, AD – 568 ± 188 pg/mL), h-tau (controls – 215 ± 73 pg/mL, MCI – 488 ± 203 pg/mL, AD – 867 ± 378 pg/mL), and p-tau (controls – 37 ± 10 pg/mL, MCI – 66 ± 22 pg/mL, AD – 102 ± 27 pg/mL) exhibited highly significant shifts in the groups. It might be important to highlight that the Con group (CSF) is referred to as the cognition factor, and therefore this population was majorly non-dementia patients of the Hospital. An overview of the control CSF is given on the Supplementary Figure 1. We would like to make a side note, that blood samples of the current study were given by other individuals, of which dementia status was checked as negative.

Additionally, we have provided table of unnormalized concentration means and standard deviations for metabolites measured in CSF and blood serum (Supplementary Tables 1, 2). A graphical representation of CSF spectral data with performed annotations is given in Supplementary Figure 2. The spectral annotation of polar metabolites (using B.I. Quant-PS™ annotated metabolites, Supplementary Figure 3) is adapted to here from an earlier communication of our group (Rössler et al., 2022).

### Control, MCI, and AD patients can be distinguished by analysis of variance (ANOVA) and by Pearson correlations of measured CSF and serum metabolites

**FIGURE 1:**
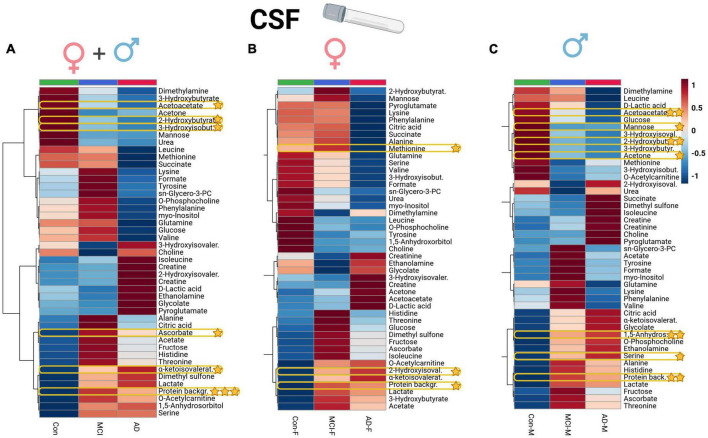
Heatmap overview plot (**A** - whole cohort, **B** - female participants, **C** - male participants) of the CSF metabolite averaged levels changes between the three groups – Alzheimer’s disease, controls and mild cognitive impairment (AD; *n* = 29, Con; *n* = 20, MCI; *n* = 22) in the CSF cohort: Each line represents a concentration change (from + 1 to –1, scaled) from the three-groups average reading. Also, each line is clustered together with other lines that were exhibiting a similar pattern in concentration changes. The Ward clustering allows to put together metabolites that displayed a similarity in averaged group changes, that could be used later in the data interpretation. In this figure, it is possible to highlight several clusters, where an averaged sample concentration was the highest. Each box is representing a group mean value in a form of color gradient (red—high concentration, blue—low concentration). AD, Alzheimer’s disease; MCI, mild cognitive impairment, Con, control subjects; CSF, cerebrospinal fluid. Significance levels: *p ≤ 0.05, **p ≤ 0.01, ***p ≤ 0.001.

**FIGURE 2:**
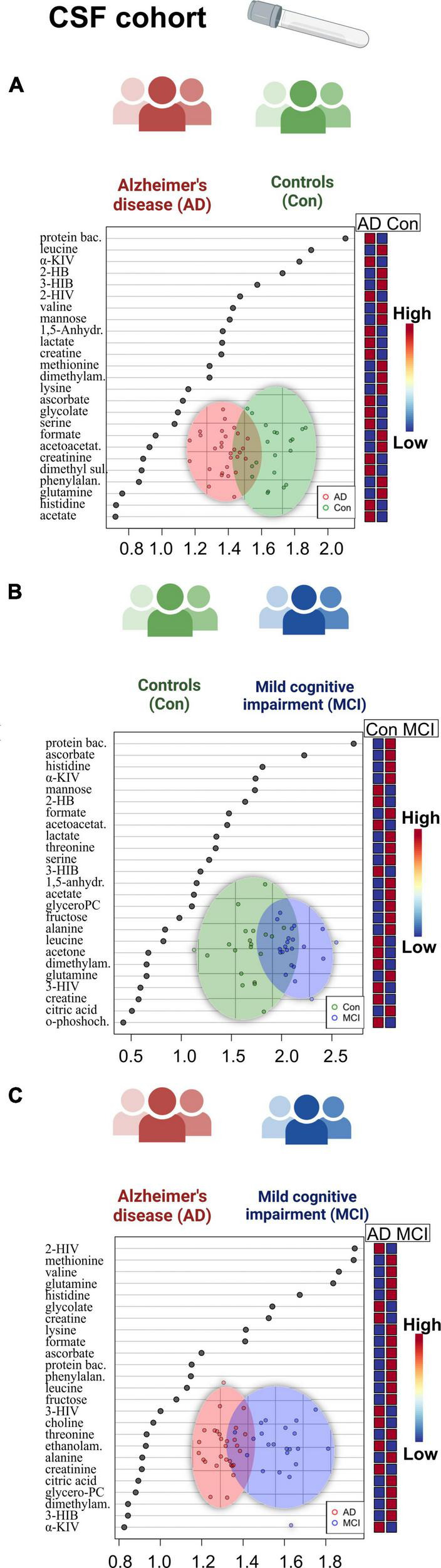
Overview of regression analysis oPLS-DA score and variables importance in projection (VIP) score plots of the CSF metabolites between Alzheimer’s disease (AD), controls (Con) and mild cognitive impairment (MCI) (AD; *n* = 29, Con; *n* = 20, MCI; *n* = 22) in the CSF cohort: **(A–C)** oPLS-DA and VIP score plots of the CSF metabolites between the groups AD and Con, MCI and Con; AD and MCI. In the VIP scores plot each box is representing a group mean value in a form of color gradient (red—high concentration, blue—low concentration). PC, phosphocholine; AD, Alzheimer’s disease; MCI, mild cognitive impairment; Con, control subjects; CSF, cerebrospinal fluid.

Univariate ANOVA analysis identified several alterations in clinical biomarkers and metabolites. In CSF aliquots, we discovered significantly altered changes of background protein content (*p* < 0.001), 2-hydroxybutyrate (2-HB), alpha-ketoisovaleric acid, ascorbate, 3-hydroxyisobutyrate (3-HIB), and acetoacetate. Whereas, acetoacetate and 3-HIB were decreasing in both AD and MCI (Supplementary Table 3; Figure 1A). The *t*-test analysis results of the CSF aliquots from AD and MCI patients revealed three significant metabolites – 2-HIV, valine (a BCAA), and creatine (Supplementary Table 4; Figure 2).

**FIGURE 3:**
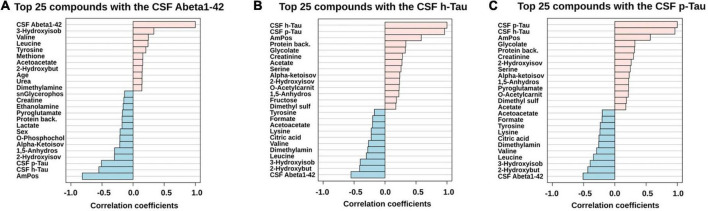
PatternHunter (Pearson correlation plots) for the CSF biomarkers Abeta **(A)**, h-Tau **(B)**, and p-Tau **(C)** with NMR-based metabolomics in CSF when comparing Alzheimer’s disease (AD) with controls (Con) and mild cognitive impairment (MCI): Each plot is a graphical representation of the top 25 variables. Positive correlations (up to 1.0) are highlighted in red, negative in blue (down to –1.0). CSF, cerebrospinal fluid.

For further statistical interpretation, we used one-variable-focused overviews of the highly correlated variables via Pearson correlations (pattern hunter). This overview approach was applied to Abeta1–42, h-Tau, and p-Tau correlations retrieved from the CSF cohort (Figure 3).

**FIGURE 4:**
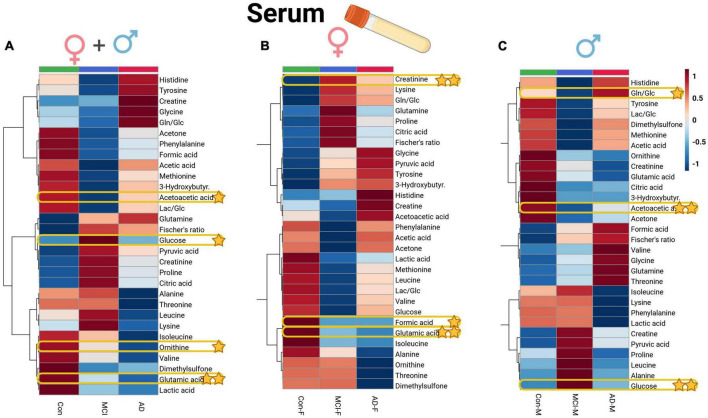
Heatmap overview plots [**(A)**–whole cohort, **(B)** female participants, **(C)** male participants] of the serum averaged levels of metabolites between the three groups – Alzheimer’s disease, controls and mild cognitive impairment (AD; *n* = 26, Con; *n* = 29, MCI; *n* = 21) in the serum cohort: Each line represents a concentration change (from + 1 to –1, scaled) from the three-groups average reading. Also, each line is clustered together with other lines that were exhibiting a similar pattern in concentration changes. The Ward clustering allows to put together metabolites that displayed a similarity in averaged group changes, that could be used later in the data interpretation. In this figure, it is possible to highlight several clusters, where an averaged sample concentration was the highest. Each box is representing a group mean value in a form of color gradient (red – high concentration, blue – low concentration). AD, Alzheimer’s disease; MCI, mild cognitive impairment; Con, control subjects. Significance levels: *p ≤ 0.05, **p ≤ 0.01.

In the case of serum metabolite data (Supplementary Table 5), glutamic acid (glutamate, *p* = 0.01) and ornithine decreased in the AD and MCI groups, while glucose showed an increase in the MCI group, and acetoacetic acid (acetoacetate) depleted in dementia individuals. Significant variables of the ANOVA could be directly compared to the whole dataset of profiled compounds within the averaged heatmap plots of the three patient groups in the serum cohort (Figure 4A). The serum heatmap plot provides an overview of the depletion of ornithine as well as BCAAs (isoleucine and valine), glutamic acid, and lactic acid. Interestingly, the aromatic amino acids histidine and tyrosine instead were elevated in the AD serum group.

**FIGURE 5:**
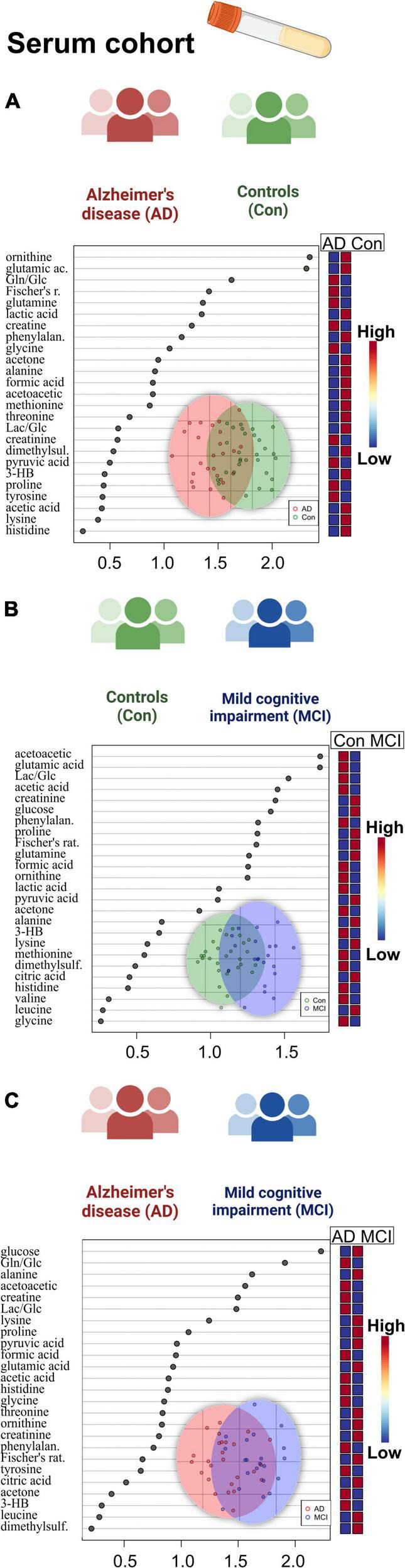
Overview of regression analysis oPLS-DA score and variables importance in projection (VIP) score plots of the serum metabolites between Alzheimer’s disease (AD), controls (Con) and mild cognitive impairment (MCI) (AD; *n* = 26, Con; *n* = 29, MCI; *n* = 21) in the serum cohort: **(A–C)** oPLS-DA and VIP score plots of the serum metabolites between the groups AD and Con, MCI and Con; AD and MCI. In the VIP scores plot each box is representing a group mean value in a form of color gradient (red – high concentration, blue – low concentration). AD, Alzheimer’s disease; MCI, mild cognitive impairment; Con, control subjects.

We also performed a comparison of statistical significance for the metabolic data shown in line with metadata entries. Therein, acetoacetate in the serum dataset was found to be significantly lowered, while glucose level rose (*p* < 0.05) in the blood of MCI-dementia patients (Supplementary Table 6; Figure 5).

**FIGURE 6:**
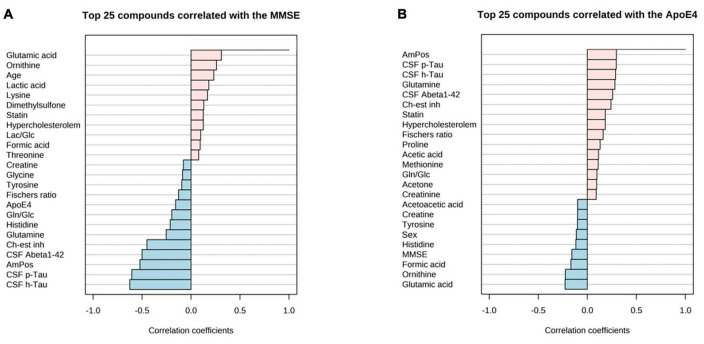
PatternHunter (Pearson correlation plots) for MMSE **(A)**, ApoE4 **(B)** with NMR-based metabolomics in blood serum when comparing Alzheimer’s disease (AD) with controls (Con) and mild cognitive impairment (MCI): Each plot is a graphical representation of the top 25 other variables that were displaying the highest absolute correlation values (both positive, up to 1.0, and negative, down to –1.0). Correlations from the study were highlighted (positive correlations marked red, negative – blue). MMSE, mini-mental state exam. ApoE-4 – amount of (0–2 copies) of the apoE-4 allele.

Correlation of MMSE score and ApoE4 levels with metabolite data revealed the following findings (Figure 6): MMSE score displayed positive slight correlation coefficients with glutamic acid and ornithine; while the ApoE4 correlations had the positive correlations observed for: glutamine, cholinesterase inhibitor medication (ch-est inh), statin and hypercholesterolemia.

### Alterations in valine and fructose metabolism in cerebrospinal fluid are characteristic discriminators between AD and MCI

A set of discriminant statistical approaches, including the *t*-test, were used to analyze two-group variations between dementia patients and cognitively healthy individuals. In addition to the metabolites which were found significant before in the three-group ANOVA comparison, the AD-MCI comparison now showed significantly reduced mannose and increased 2-hydroxyisovalerate (2-HIV) in AD. Additionally, we determined a set of metabolic variables significantly altered in the sub-cohort of CSF with the available CSF biomarkers entries (Supplementary Table 4). As a result, we discovered significantly lowered CSF protein content and 2-HIV levels in AD patients who were majorly amyloid positive (AmPos) compared to the MCI patient group.

When we proceeded to the multivariate oPLS-DA analysis (Figure 2), we saw reduced BCAA (valine) in the AD patient group. There was also a rise in 1,5-anhydrosorbitol, and lactate levels, and a decrease in mannose, three of which are markers of altered fructose metabolism. Finally, we were able to find histidine and ascorbate (vitamin C) both elevated in the patients with mild cognitive impairment based on the MCI-Con two-group comparison (Supplementary Table 4).

### CSF fructose and CSF protein correlated with clinical AD biomarkers

We used *t*-test analysis to compare metabolomic, lipoprotein, and metadata characteristics between the AD-dementia and mild MCI patient groups. Based on the available dataset entries from the serum cohort in the two-group discrimination, it was discovered that the MMSE score performed best (*p* < 0.001), Supplementary Table 6. There were also significant alterations in the glutamine/glucose ratio (Gln/Glc, higher in AD) and glucose levels (higher in MCI).

Remarkably, there were more substantial dementia-defined alterations from the oPLS-DA score and VIP, the limited size cohort was used, in the CSF analysis (Supplementary Figure 4). Within the regression model oPLS-DA, only MMSE score and CSF h-Tau levels were relevant. In CSF, the clearest metabolic changes were from fructose and CSF protein content. The oPLS-DA analysis score graphs demonstrated a qualitative cluster separation for the CSF patients’ samples.

### Metabolomic profiles of CSF and serum reveal sex-specific alterations

Applying a correlational and regression model statistic (Supplementary Figure 5) that included age and sex metadata entries of the whole cohort, we assessed the trend and association of metabolomic parameters to the sex factor, mainly of CSF (Figures 1B, C) and serum BCAAs, lactate from CSF, and serum creatine (Figures 4B, C). By using the sparse regression model, we may observe mild trends of sex-specific differentiations among the AD-MCI-Con groups. As such, we may highlight those that contributed the most to the discrimination of the analyzed clusters. Female AD and MCI individuals were highly different via CSF methionine levels, while metabolomic differences in AD-Con female groups were well described with serum glutamic acid, creatinine, and formic acid. Interestingly alternative characteristics could be seen for the male subjects of the study. Herein, we were able to determine that male controls had a largely higher level of CSF 2-hydroxybutyrate. Serum levels of male sub-groups would allow to differentiate MCI males group via elevated serum glucose and lowered acetoacetic acid levels from their AD and Con counterparts. We evaluated sex-based changes by means of a regression analysis of the (PLS-DA) VIP values exclusively for Con, AD, and MCI (Supplementary Figure 6). Among the CSF samples strong connections could be seen between the sex factor and CSF lactate also serum creatinine.

Furthermore, we were able to further discover a substantial elevation of serum creatine in AD female participants (compared with AD males); serum creatinine and creatine levels were higher in female controls (compared with male controls) by analyzing the statistical summary of the current data, which is shown in Supplementary Table 7. None of the interesting outcomes of the whole-cohort CSF (serum metabolites were insignificant) and sex-based entries substantially (by *p*-value via ANOVA) contrasting the two different patient groups were brought to the context of the sex-based bias investigation (Supplementary Table 8).

After the FDR adjustment, many variables of the cerebrospinal fluid (Supplementary Table 9), namely valine and lactate, were shown to be considerably correlated with the sex factor and retained their significance. This indicates that the entire CSF data pool is not yet big enough to be statistically independent of the metadata parameters of the patients since group sizes within this study were rather modest.

### Area-under-curve analysis revealed valine serum/CSF ratio as a significant parameter in AD/MCI discrimination

We also looked at the CSF-to-serum ratios of metabolic values for the following 22 metabolites: 3-hydroxybutyrate (3-HB, 3-hydroxybutyric acid), acetate (acetic acid), acetoacetate (acetoacetic acid), acetone, alanine, citric acid, creatine, creatinine, dimethyl sulfone, formate (formic acid), glucose, glutamine, histidine, isoleucine, lactate (lactic acid), leucine, lysine, methionine, phenylalanine, threonine, tyrosine, valine. Valine serum-to-CSF ratio was significant (Supplementary Figure 7) in discriminating the AD and MCI patient groups based on the univariate AUC-ROC analysis (Supplementary Table 10).

## Discussion

### Lower levels of ketone bodies in CSF and serum may induce AD-driven dementia

We discovered distinctive alterations in the established AD CSF biomarkers which were consistent with previous studies (Lewczuk et al., 2020). Adding metabolomics data to these findings can potentially resolve AD-driven processes in the human body that have not been elucidated so far.

Hypercholesterolemia and hyperglycemia are dangerous AD pre-conditions, potentially aggravating metabolic conditions in the brain (Kivipelto, 2001; Crane et al., 2013). A ketogenic diet could protect the brain from excessive glucose levels linked to AD-like conditions and diabetes (Yancy et al., 2005; Paoli et al., 2014; Zilberter and Zilberter, 2017). Additionally, a ketogenic diet given to AD and MCI patients was reported to improve memory and compensate for diminished brain glucose uptake (Cunnane et al., 2016).

Ketone bodies serve as an alternative energy source in case of glucose deficiency in the dementia brain (Hertz et al., 2015). We found no evidence of reduced CSF and serum glucose in the AD and MCI groups, however have to be aware that serum samples were not strictly sampled always under fasting conditions. On the contrary, the MCI group showed greater blood glucose levels. We also had seen no significant changes in patients’ CSF glucose levels. However, glucose has a swift turnover. For human subjects, it is impossible to standardize sampling preconditions (like fasting) to 100%.

From the standpoint of brain energy supply by ketone bodies, of which acetoacetate (acetoacetic acid) was significantly decreased in both CSF and serum measurements of patient cohorts. Both 3-hydroxybutyrate and acetoacetate potentially have neuroprotective properties due to the reduction of ROS effects (Yang et al., 2019). The reduced ketone body concentrations could suggest a reduced neuroprotective effect and brain energy deficit. Acetoacetate (acetoacetic acid) was nominated to be among the key CSF metabolites of the cognitive decline state (Peng et al., 2020).

Therefore, we propose that dementia is caused by a breakdown of brain energy balance, which leads to specific metabolic alterations in both CSF and serum. Furthermore, we suppose that the imbalance in glucose consumption can also be seen in the AD group, as seen by a slight increase in lactate CSF concentrations, which is to previous findings (Redjems-Bennani et al., 1998; Nakano et al., 2017). Another notable metabolite shift was observed for fructose, which was higher in the MCI group and might be associated with the polyol pathway. Wherein, high fructose and 1,5-anhydrosorbitol (slightly elevated in the AD group) were previously reported to be a significant alteration in a murine model of Parkinson’s disease (Hauser et al., 2017).

### Reduced amounts of branched-chain amino acids in CSF and serum link to metabolites associated with valine degradation

Higher blood BCAAs levels negatively influence tryptophan uptake into the brain at the blood-brain barrier. Therefore, BCAAs could affect serotonin production and signaling and thus, could potentially be linked to Abeta plaque formation (Larsson and Markus, 2017). Yet, some reports have shown lower BCAAs levels that were associated with AD (Ikeuchi et al., 2022; Siddik et al., 2022). Therefore, we believe that these changes could be an indication of cohort specificity. Longitudinal increase of BCAAs uptake was also believed to increase AD-driven dementia severity significantly (Larsson and Markus, 2017). In an animal model of AD, dietary supplementation of valine, leucine, and isoleucine led to higher tau levels (Tournissac et al., 2018).

An additional metabolite that can be linked to the described BCAAs metabolism alterations (Liebich and Först, 1984), as well as ketogenesis, is 2-hydroxyisovalerate (2-HIV), which was significantly higher in the AD group. Further, upregulated creatine in the AD group could indicate altered guanidinoacetate metabolism, among other energy-related pathways (Persky and Brazeau, 2001).

Another interesting altered metabolite is alpha-ketoisovaleric acid (ketoisovalerate), which was significantly higher in cerebrospinal fluid analytes of dementia patients. Ketoisovalerate is involved in the valine and leucine catabolic pathway, implying a change in the BCAAs levels in CSF of cognitively impaired individuals. It is worth noting that the *t*-test analysis revealed both ketoisovalerate and 3-HIB to be statistically significant only for the AD-Con comparison. The ketoisovalerate alterations in CSF could also explain changes in 3-hydroxyisobutyrate (3-HIB). 3-HIB is a metabolite that is closely associated with the valine degradation pathway (Wanders et al., 2012).

### Abeta and tau highly correlate with CSF metabolites, while MMSE and APOE4 correlate with serum glutamine and glutamic acid

In addition to using the established CSF Abeta and tau biomarkers for the diagnosis of AD, we were also provided with the clinical MMSE score, ApoE4 alleles count, and cholinesterase inhibition therapy positive status. These biomarkers could provide a categorical ranking of a patients’ state of dementia. As a result, identifying the metadata parameters to the main experimental tables in both CSF and serum sub-cohorts has become an important goal.

A set of three CSF metabolic parameters (glycolate, CSF protein content, acetate) was identified to positively correlate with CSF tau levels. Several studies indicate a potential link between AD and the glycation process of proteins and the formation of advanced end glycation products (AGEs) (Shuvaev et al., 2001; Ahmed et al., 2005; Yamagishi et al., 2005; Prasad, 2019). The measurement of the CSF AGEs formation due to glucose utilization is reserved for future investigations, as it has been previously registered at relatively low concentration levels in ELISA tests (Monacelli et al., 2014). Despite the low statistical significance, CSF glycolate could be potentially linked to the AGEs (Dutta et al., 2007).

Interestingly, higher CSF protein content as measured by NMR was closely associated with tau protein amounts in both AD- and MCI-diagnosed patients. The CSF protein content variable measured via NMR metabolomics presents the total CSF protein content, e.g., albumin content in cerebrospinal fluid aliquots. As a result, we presume that the observed increase in total protein levels may be linked to a slower renewal of cerebrospinal fluid in patients (Mesquita et al., 2012; Marques et al., 2013; Krzyzanowska et al., 2015).

Higher CSF acetate in dementia patients demonstrates the same trend for the dementia patients described previously (Laakso et al., 2015; Vignoli et al., 2020). Acetate takes part in the formation of the brain tissue metabolite N-acetylaspartate (NAA), Coenzyme A (CoA), and a key neurotransmitter O-acetylcholine.

Earlier on, we described the significance of BCAAs (leucine and valine) and 3-hydroxyisobutyrate in cerebrospinal fluid aliquots. Interestingly, these variables had demonstrated negative correlations to tau protein levels and positive – to the CSF Abeta levels. Therefore, we could support that CSF AD and MCI dementia patients have a high significance of BCAA metabolism alongside valine degradation pathway to take place and become a potential target in SOP CSF screening within the future studies of the neuropathology of Alzheimer’s.

Correlational PatternHunter plots (serum) narrowed down glutamic acid and glutamine as highly changing in dementia. We were also able to detect that ornithine levels were in lower amounts together with serum glutamic acid, while higher glutamine serum levels were found in both patient groups. Blood glutamine and glutamate level changes were reported in the context of metabolic disruption in AD and MCI patients’ blood analytes (Wang et al., 2014; Nuzzo et al., 2021). Our results showed serum ornithine depletion in the AD and MCI groups. A strong negative correlation of ornithine toward Abeta in both CSF and serum for AD patients was previously determined (van der Velpen et al., 2019). Therefore, the polyamine pathway produces a considerable contribution to altering the metabolomic profile of early- and late-onset AD patients. One study revealed that the polyamine pathway is the leading factor for discriminating MCI metabolic profile when compared against controls in human blood samples (Graham et al., 2015).

### Stronger changes of BCAAs and lactate among females could indicate that AD-driven dementia is accelerated in female patients

Notably, the ranking variables of CSF lactate in the VIP scores plot were shown to have a significantly greater average concentration in the CSF of female AD and MCI patients than in males of the sample phenotype. In elderly, CSF lactate is reported to be interacted with CSF glucose but not with the sex factor (Nakano et al., 2017). However, we believe that the statistical regression model also does not highly prioritize lactate against other upcoming metabolites. Considering serum creatinine changes based on the sex factor, this has been vastly investigated and standardized tools of creatinine determination allow to narrow down blood concretions of that metabolite down to sex, age, and also factors like ethnicity (Delanaye et al., 2017).

Within the scope of the present investigation, we are able to emphasize the observed creatine levels high in the female part of our serum cohort with a specifically high positive correlation with the sex factor (and we saw some significance between creatine in CSF to the sex factor of the whole cohort of patients) that may indicate the differentiation of sex-based blood creatine differences (higher creatine in women) as reported in Rist et al. (2017). For a clinical cohort of a similar scale (Ruoppolo et al., 2014), it was also previously shown that glycine levels could appear significantly higher in women than in men serum samples, as we identified in our whole-cohort high positive glycine-sex correlation.

Related to the AD-related anomalies, we were able to see some sex-based differentiations in phenotype via the regression model approach applied, i.e., CSF methionine elevation that was highly characterizing the female MCI patients versus their female-AD counterparts (had lowered CSF methionine). It is possible that as of an AD-potential transitionary stage, the higher female-MCI over AD methionine in CSF, where MCI is generally more often occurrence in women (Au et al., 2017), could be placed in parallel to the higher CSF methionine among psychotic disorders patients (Regland et al., 2004). Patients with psychotic diseases, at least during a period of acute exacerbation, are often in a condition of abnormal one-carbon metabolism, for which there was no explanation for the rise in methionine. However, the methionine pathway distress is a risk factor for cognitive decline and dementia, including AD (Dayon et al., 2017). Previous research supports the notion that L-dopa promotes methionine cycle abnormalities (Andréasson et al., 2017). In older individuals, cognitive function and CSF measurements of AD pathology are dependent on one-carbon metabolism, which plays a major role in the production of several essential compounds (Dayon et al., 2017). Overall, this concludes that for some portion of female AD-MCI groups from our current study, the sulfuric amino acid methionine and its closely associated metabolites and functions could be severely altered, yet for further confirmation a new collection of CSF samples of MCI and AD patients would be required.

Within the male part of the CSF cohort investigation of existing sex-based bias in AD and MCI against the healthy controls, we were able to detect unique findings of CSF 2-hydroxybutyrate being lowered in male dementia (AD, MCI) patients compared to controls. Similarly, CSF and also serum acetoacetate, and a number of other regression model-significant parameters were attributed to the male AD-Con-MCI 3-group comparison. Additionally, 2-hydroxybutyrate has been identified as an early marker for impaired glucose regulation and indicates underlying metabolism of oxidative stress (Gall et al., 2010). Moreover, some studies also showed that 2-hydroxybutyrate could reflect changes in the progression of dementia (Nagata et al., 2018; Teruya et al., 2021). For instance, Multiple sclerosis and Devic’s disease were characterized by higher 2-hydroxybutyrate via NMR-based metabolomics of a CSF cohort (Kim et al., 2017).

In general, the observed correlations of BCAAs for the female-characteristic sex component of the both CSF and serum patient cohorts are rather rich with significance (as measured by raw *p*-value) and are similar to lipoprotein panel bias toward the sex factor reported for the same utilized serum patients’ cohort recently (Berezhnoy et al., 2022). However, the sex-based differences in BCAAs concentrations are known and could be induced by, e.g., a diet intervention.

Of note, we believe that the current study had been influenced by an imbalance in terms of sex between the two patient groups analyzed, except the AD group, as there were much fewer proportions of female patients in these two patient groups (CSF Con, CSF MCI, and Serum MCI). As a result, it is possible that a male-based phenotypic influence was the most prevalent.

**FIGURE 7:**
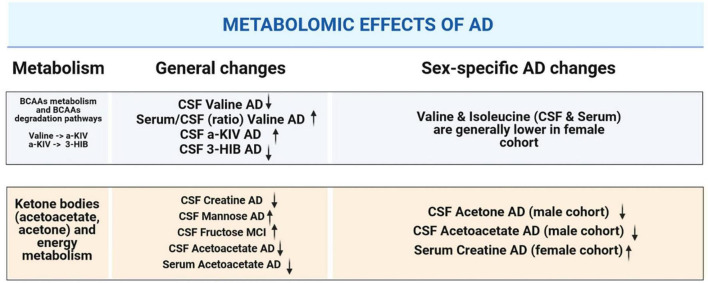
Graphical summary illustrating alterations in CSF and serum metabolites that are significant: in the context of dementia-driven and sex-based changes.

Overall, our NMR metabolomic data for serum and CSF revealed some noteworthy differences within the studied cohorts. Therefore, we provide a graphical summary of the patient groups’ comparison (Figure 7).

### CSF and serum valine altered for 39 patients who had given CSF and serum aliquot

The 39-patient-based comparison of both biofluids was performed to see whether there were any novel cohort-specific between AD and MCI dementia stages. Only metabolites that were identified in both biofluids were applied for the *t*-test analysis. Wherein, lysine and formate in CSF showed significantly lower concentrations in the AD specimen. Valine had an altered CSF-to-serum ratio that had been a single statistically and AUC-significant entry.

Within this study, the current finding of valine (serum/CSF) is represented as a well-performing AD-MCI patient cohort discriminator, that could be further tested within the context of future dementia investigations.

### Strength and limitations of the study

The implementation of this NMR-based metabolomics approach to human biofluids of dementia patients using a strict SOP has been found to be a major strength of the current study. Primarily, we are able to initially minimize sample preparation inaccuracies and concentration shifts in analytes. Secondly, with the possibility of wider implementation of the same SOP to other blood (plasma, serum) and cerebrospinal fluid samples, researchers would obtain an advantage of a direct comparison of summarized data matrixes including meta-cohort analysis. Indeed, the overall number of CSF and serum samples (Table 1) inside this study is only modest, so future investigations should make use of larger cohorts, if possible. That likely will help to identify additional metabolic alterations, which fell below the statistical threshold within this study.

The sex-specific analysis, herein, is resulting in a lower statistical significance due to the overall segmentation of a limited cohort size. As this study was based on a retrospective biobank cohort unfortunately no more samples could be obtained. Our results still show sex-specific trends which we hope to be validated in future investigations having access to larger cohorts.

## Conclusion

Our quantitative NMR-based metabolomics investigation of CSF and serum aliquots from AD and MCI patients and individuals with cognitively normal function identified several metabolite alterations. The results were obtained from both biofluids and were identified to be ketone bodies, branch-chained amino acids, and brain energy metabolites in CSF. On the other hand, glutamic acid, acetoacetic acid (a ketone body), and valine (a branched-chain amino acid) were considerably changed in patients’ serum metabolome. From unsupervised statistical analysis, we believe that ketone bodies-associated differences in CSF between the patient groups could provide significant input for dementia patients’ screening and could provide an additional axis of comparison of dementia progression. Within the regression model analysis, patient MMSE score and CSF tau protein levels were highly correlative in CSF with the elevation of CSF protein content in both patient groups. Wherein, higher CSF fructose and 1,5-anhydrosorbitol levels identified a potential pathological role of the polyol pathway. We also believe that another key to AD screening might be 2-hydroxybutyrate, a potential pre-diabetes condition marker as well as a metabolite demonstrating an influence of oxidative stress. Finally, we believe that the diagnostic combination of small molecule type metabolites and AD clinical biomarkers could be a first step toward establishing an expanded tool for longitudinal monitoring of dementia patients.

## Data availability statement

Mean concentrations with standard deviations for CSF and serum metabolites are provided in the Supplementary material. Raw NMR spectroscopy data will be made available by the authors without undue reservation upon request.

## Ethics statement

The studies involving human participants were reviewed and approved by the Ethics Committee of the Medical Faculty of Eberhard-Karls-University and University Hospital Tübingen (protocol code 721/2015BO2, 07.08.2019, SOP-protocol Biobank HIH-Biobank version 1.2.5, February 2020). In the CSF samples study, the protocol was approved by the local institutional review boards and ethical committees of all participating sites of the German Center for Neurodegenerative Diseases (DZNE). The study was conducted in accordance with the Declaration of Helsinki. The patients/participants provided their written informed consent to participate in this study.

## Author contributions

GB, CL, and CT contributed to the experimental design, data acquisition, and data analysis and interpretation. GB wrote the original draft. All authors approved the final manuscript.
