# Supplementary material for: Metabolomic profiling of CSF and blood serum elucidates general and sex-specific patterns for mild cognitive impairment and Alzheimer’s disease patients
Source: Front Aging Neurosci. 2023 Aug 24;15:1219718. doi: 10.3389/fnagi.2023.1219718 (PMC10483152; doi:10.3389/fnagi.2023.1219718)
Supplement: Supplementary file 1 [file Data_Sheet_1.docx]

# Supplementary Figures

**
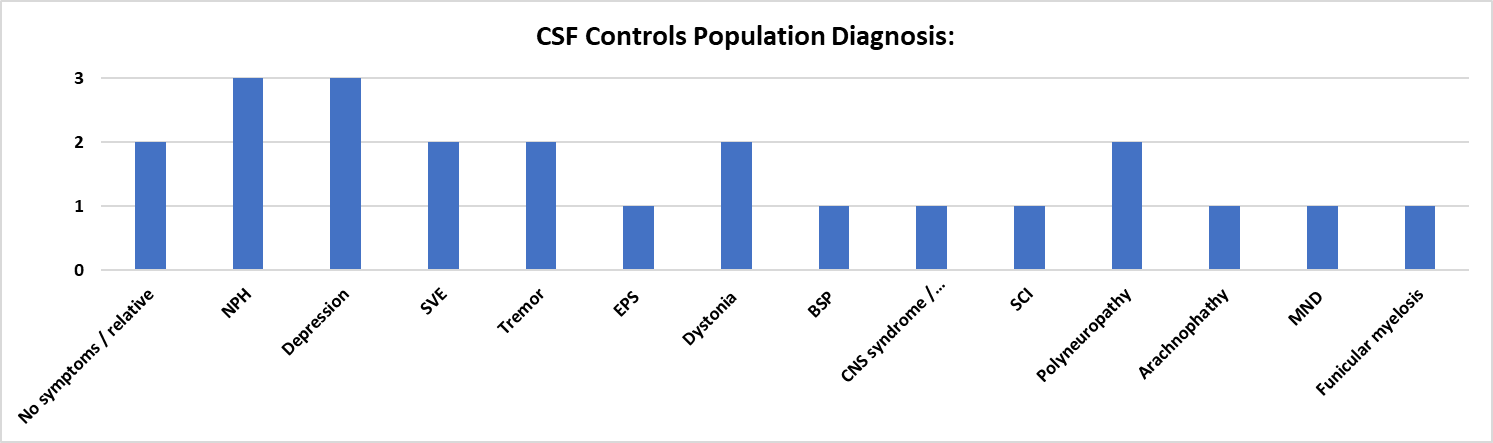
**

**Suppl. Fig. 1. Cohort depiction diagram (number of patients with the disease case) of the Con group (CSF aliquots, n = 20).** NPH – normal pressure hydrocephalus. SVE - subcortical vascular encephalopathy. EPS - extrapyramidal symptoms. BSP – blepharospasm. CNS syndrome - inflammatory diseases of the CNS (central nervous system). SCI - Spinal cord injury. MND - motor neuron disease.

**
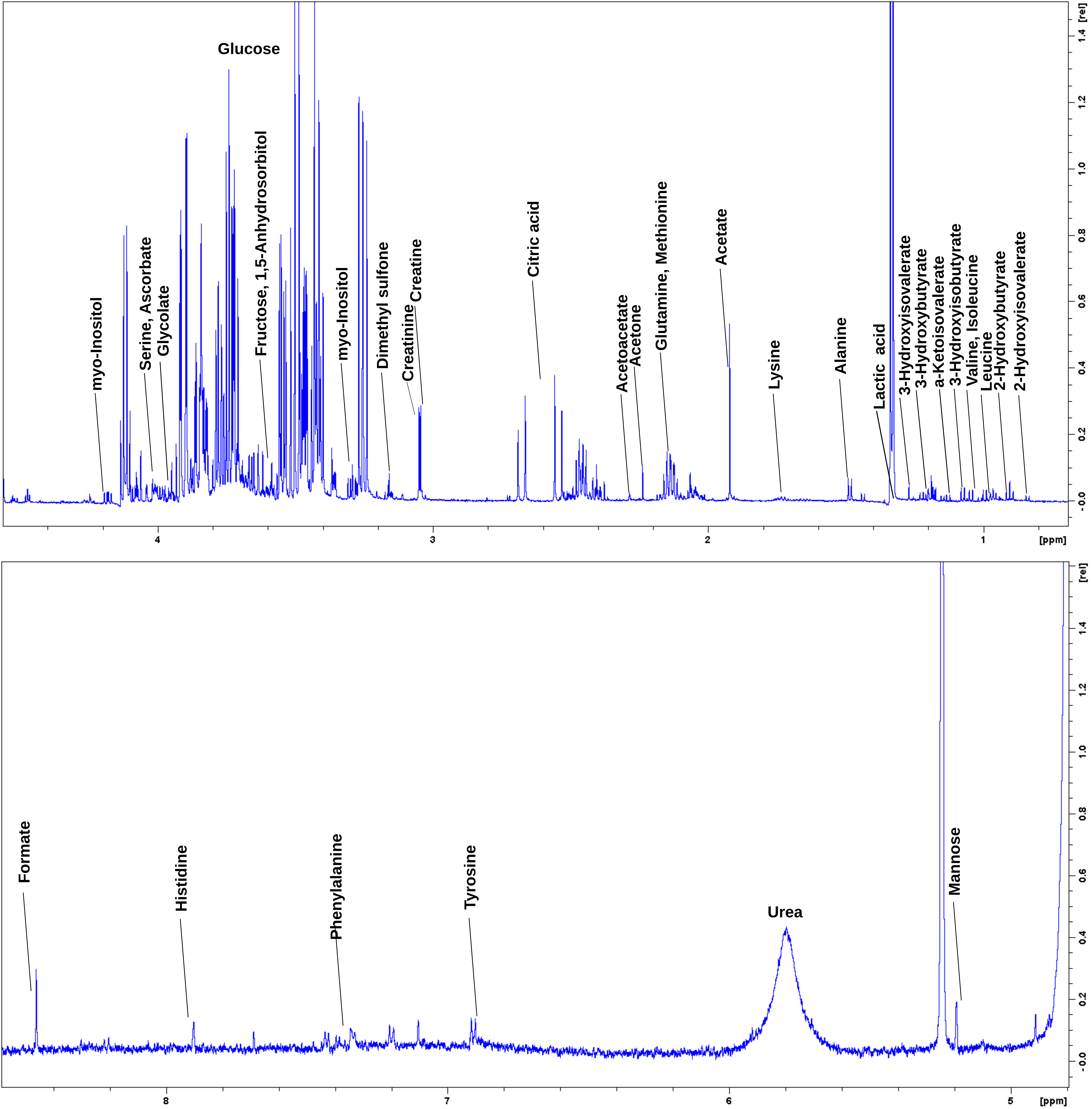
Suppl. Fig. 2. The example NMR spectrum of the CSF displaying annotated metabolites.**

**
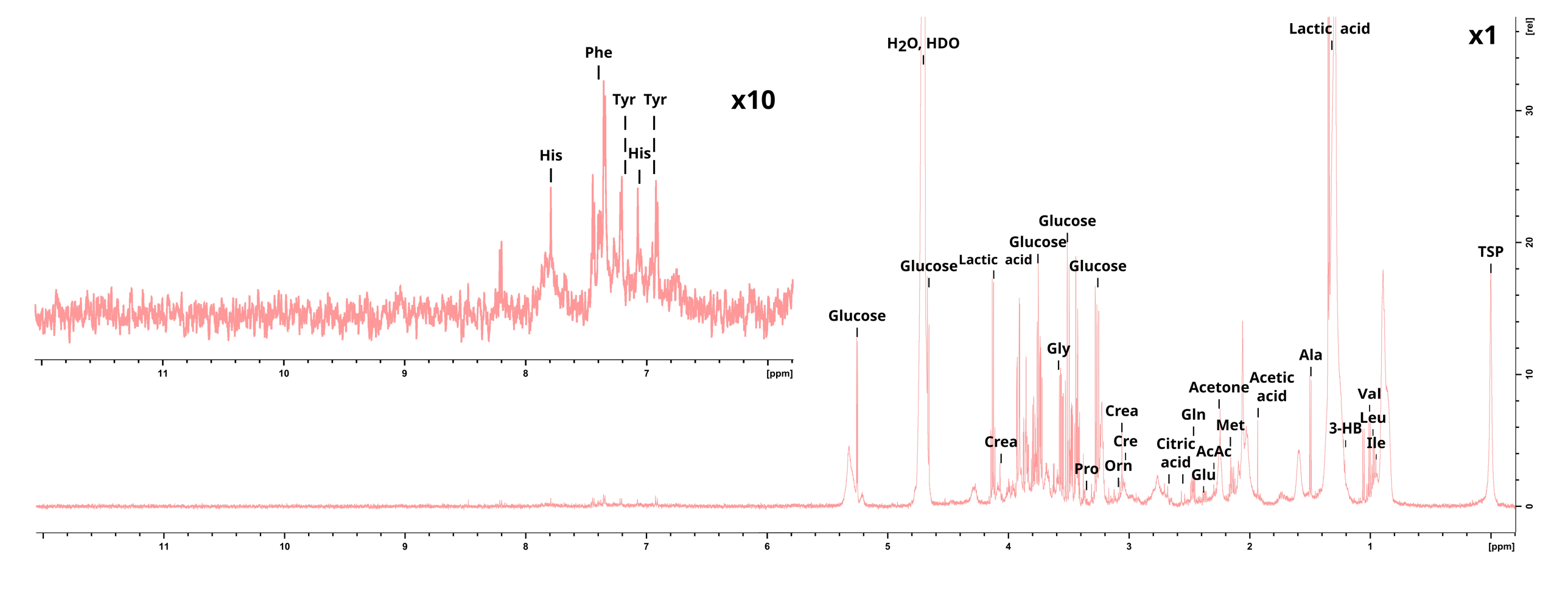
**

**Suppl. Fig. 3. The example NMR spectrum of the serum displaying annotated metabolites,** **whereas the aromatic region (from 8.0 till 6.0 ppm) was zoomed in by a factor of 10 times.**

**
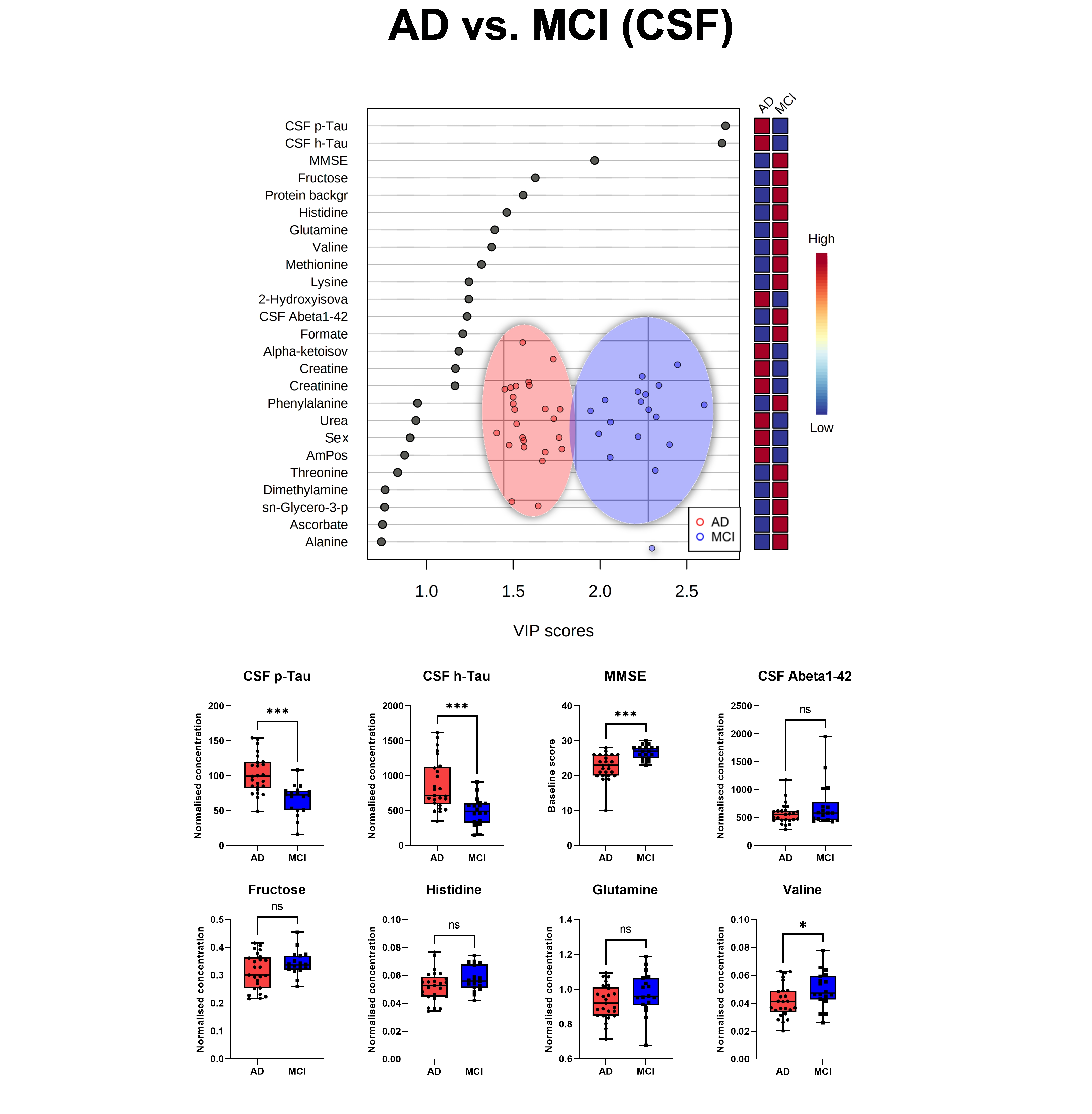
**

**Suppl. Fig. 4. Regression model analysis (oPLS-DA) results and variables in projection importance score (VIP; below some of the variables box plots are shown) based on data from CSF metabolites and metadata entries significantly (by p value) comparing two patient groups (AD; n = 25, MCI; n = 18) in the cerebrospinal fluid analytes.** VIP score is providing top-25 most significant variables that were found fit for group discrimination alongside that T-score (horizontal) axis of the regression model analysis score plot. AD – Alzheimer’s disease group. Con – control subjects. MCI – mild cognitive impairment patients. Statistical significance: ns – not significant, * p ≤ 0.05, ** p ≤ 0.01, *** p ≤ 0.001.


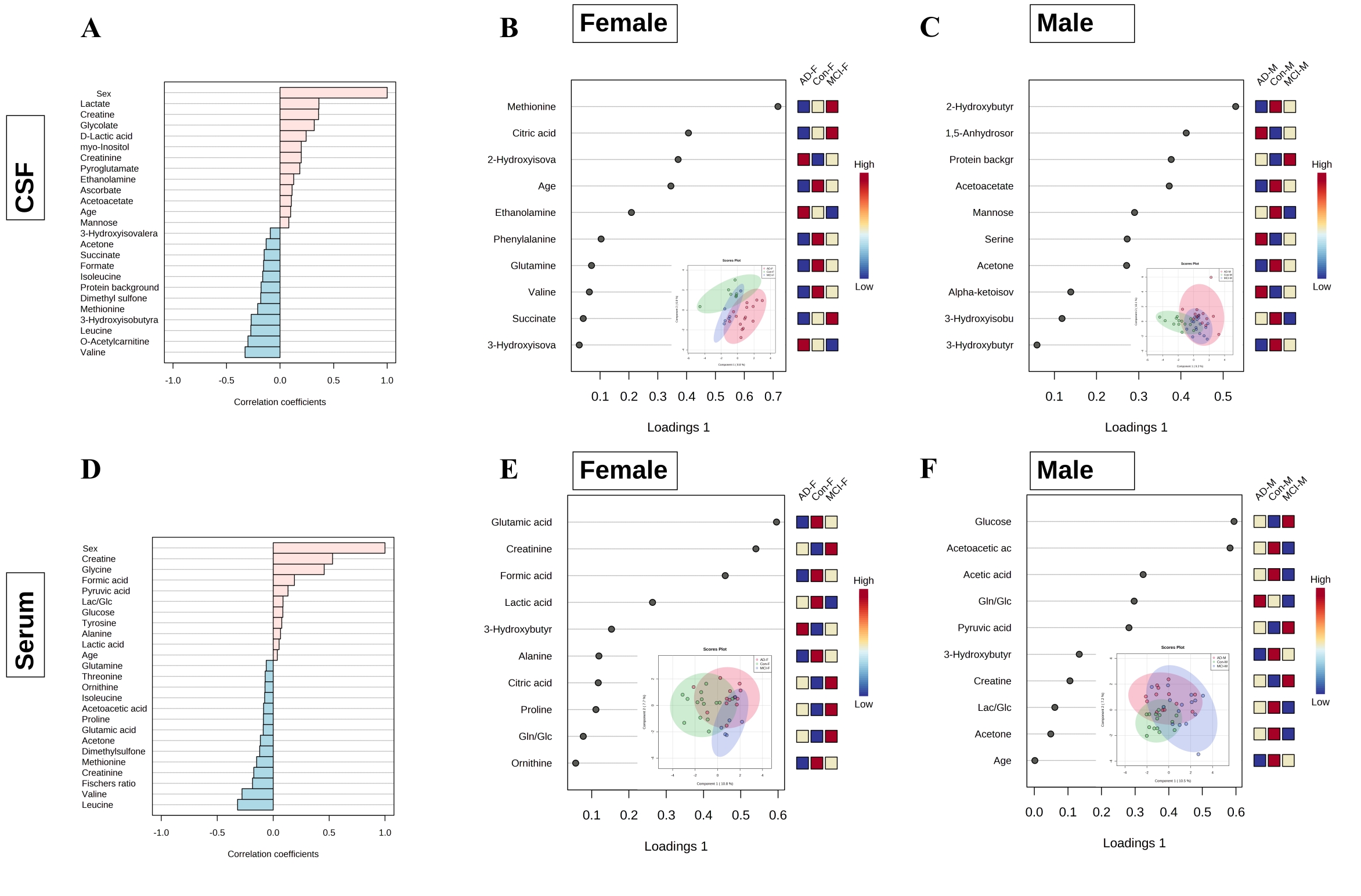


**Suppl. Fig. 5.** **Correlational PatternHunter whole-cohort analysis (Pearson correlations, A – for CSF metabolites) and multivariate analysis of the three male/female groups’ comparisons based on sPLS-DA regression model analysis of three patient group comparisons separated by sex criterion is shown (B-C – for CSF metabolites).** Also, the same way of analysis was applied to the serum data. The correlational plots (A and D, D – for serum metabolites) include the top-25 variables ranked by the absolute values of the correlation coefficient to the sex factor, which is 1 for female and 0 for male. The sPLS-DA loadings plots for the Component 1 (B, C, E, and F, where E-F – for serum metabolites) demonstrate top-10 variables that contributed to the regression model built. F–female. M–male.


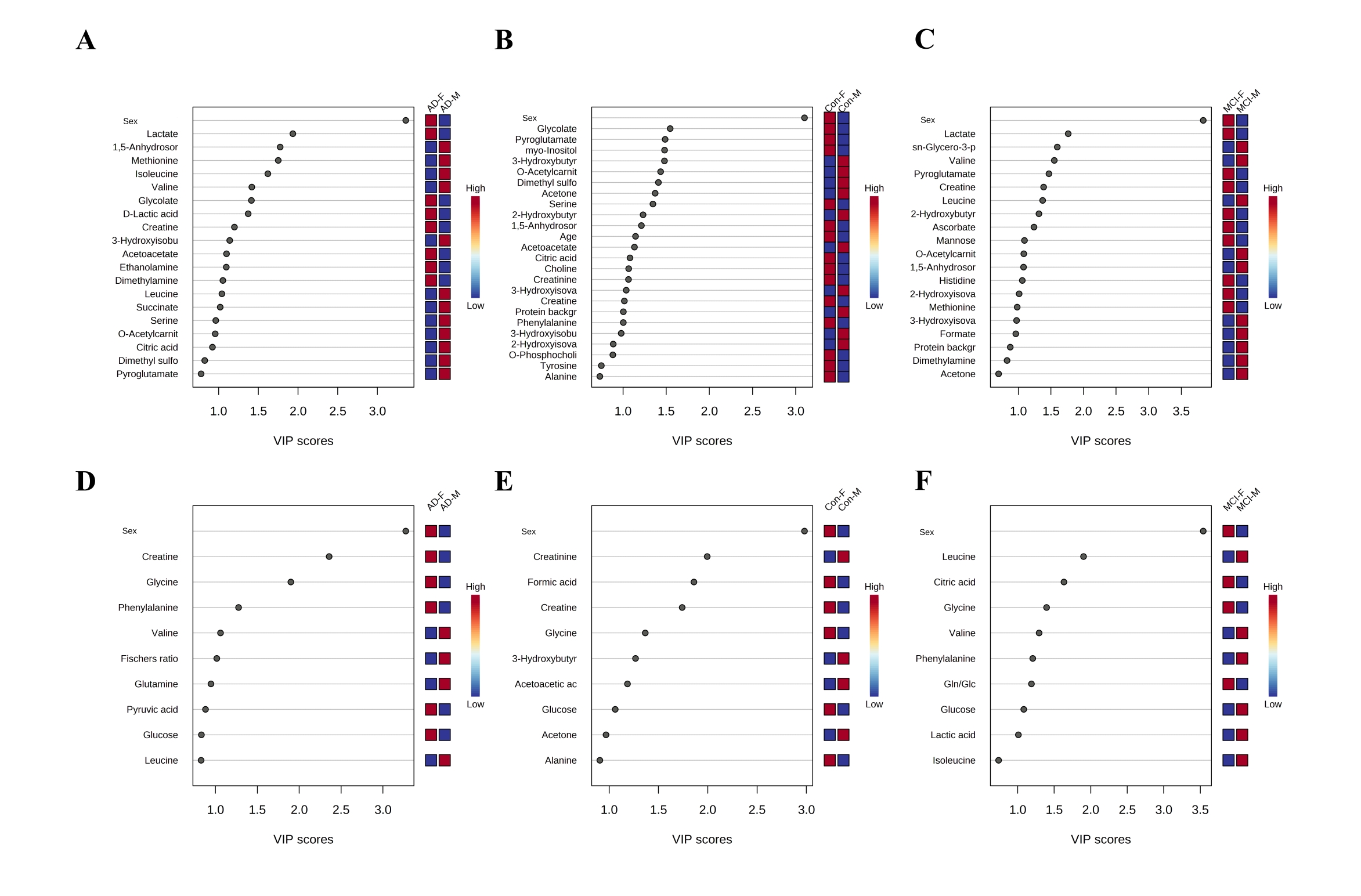


**Suppl. Fig. 6.** **Multivariate analysis of the three male-female group whole-cohort comparisons based on oPLS-DA ((Panel A – for CSF metabolites), in the cognitively control group; (Panel B – for CSF metabolites), AD patients; (Panel C – for CSF metabolites), MCI patients).** The very same analysis was performed for the serum metabolites data Panels D-F. The score plots include the VIP scores > 1.0. F–female. M–male.


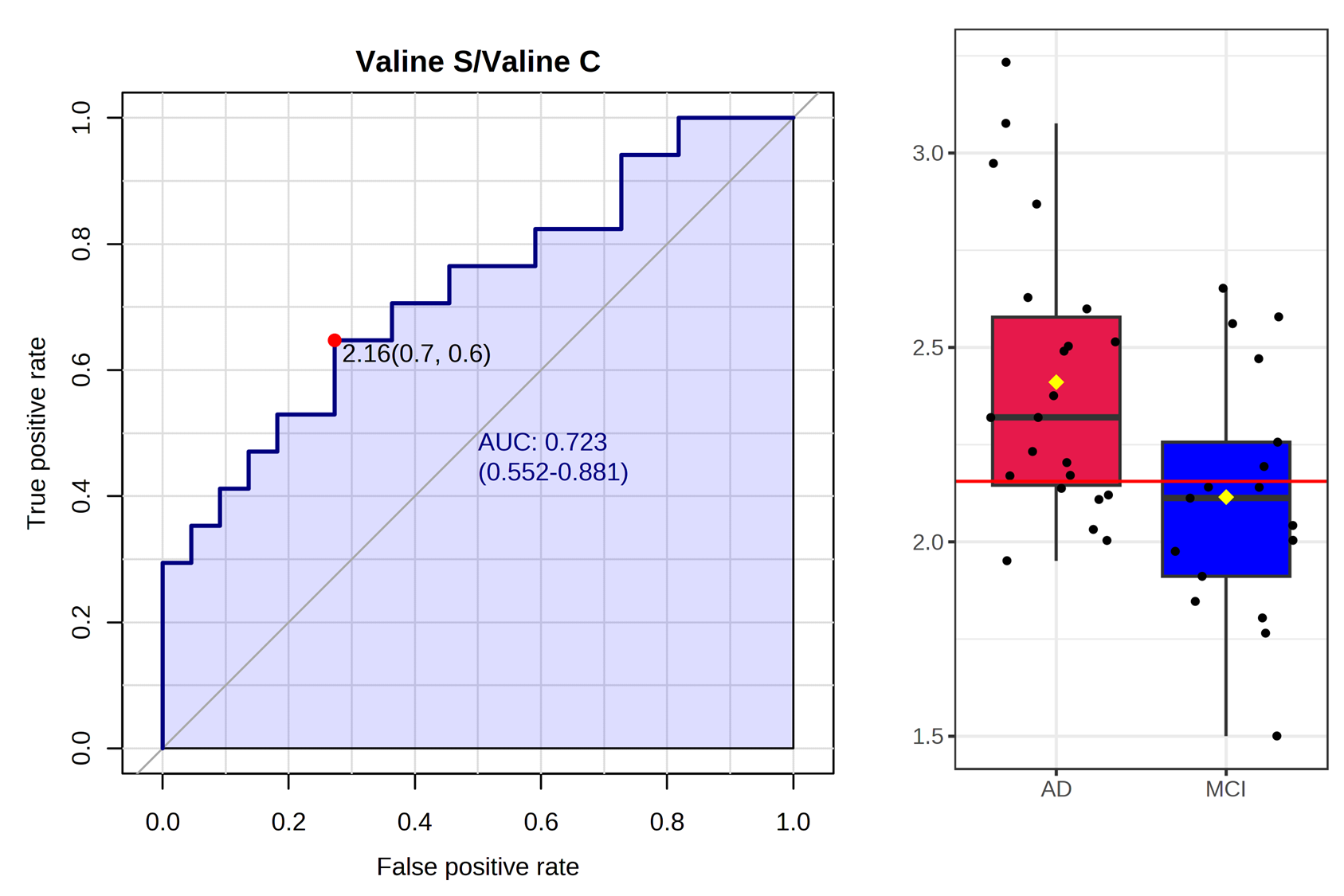


**Suppl. Fig. 7.** **Receiver operating characteristic (ROC) curves and box plot distribution:** ratio of serum-to-CSF valine levels that was identified in the studied aliquots based on the common patients’ sub-cohort (AD; n = 22, and MCI; n = 17). The comparison is based on the limited CSF-serum data sub-cohort. Significance level – (p ≤ 0.05); p = 0.0111. C – a variable from the CSF cohort dataset. S – a variable from the serum cohort dataset.

**Supplementary Tables**

| **CSF**  **Parameter (mmol/L)** | **Mean Value AD**  **(n=29)** | **Standard Deviation AD** | **Mean Value Con**  **(n=20)** | **Standard Deviation Con** | **Mean Value MCI**  **(n=22)** | **Standard Deviation MCI** |
| --- | --- | --- | --- | --- | --- | --- |
| 1,5-Anhydrosorbitol | 0.083 | 0.045 | 0.067 | 0.034 | 0.082 | 0.044 |
| 2-Hydroxybutyrate | 0.032 | 0.015 | 0.041 | 0.019 | 0.036 | 0.024 |
| 2-Hydroxyisovalerate | 0.010 | 0.010 | 0.006 | 0.004 | 0.006 | 0.004 |
| 3-Hydroxybutyrate | 0.010 | 0.008 | 0.013 | 0.020 | 0.010 | 0.007 |
| 3-Hydroxyisobutyrate | 0.016 | 0.008 | 0.018 | 0.005 | 0.017 | 0.007 |
| 3-Hydroxyisovalerate | 0.005 | 0.002 | 0.005 | 0.002 | 0.005 | 0.002 |
| Acetate | 0.035 | 0.042 | 0.033 | 0.036 | 0.039 | 0.031 |
| Acetoacetate | 0.009 | 0.007 | 0.012 | 0.011 | 0.009 | 0.005 |
| Acetone | 0.024 | 0.020 | 0.033 | 0.045 | 0.022 | 0.013 |
| Alanine | 0.055 | 0.023 | 0.051 | 0.013 | 0.062 | 0.029 |
| Alpha-ketoisovaleric acid | 0.004 | 0.002 | 0.004 | 0.001 | 0.004 | 0.002 |
| Ascorbate | 0.252 | 0.120 | 0.213 | 0.065 | 0.279 | 0.126 |
| Protein background | 0.003 | 0.002 | 0.002 | 0.001 | 0.003 | 0.001 |
| Choline | 0.004 | 0.002 | 0.004 | 0.001 | 0.004 | 0.002 |
| Citric acid | 0.200 | 0.097 | 0.190 | 0.063 | 0.211 | 0.100 |
| Creatine | 0.067 | 0.031 | 0.060 | 0.014 | 0.065 | 0.027 |
| Creatinine | 0.103 | 0.044 | 0.093 | 0.025 | 0.101 | 0.047 |
| D-Lactic acid | 0.007 | 0.004 | 0.006 | 0.002 | 0.007 | 0.004 |
| Dimethyl sulfone | 0.019 | 0.020 | 0.014 | 0.007 | 0.018 | 0.012 |
| Dimethylamine | 0.004 | 0.002 | 0.004 | 0.001 | 0.004 | 0.002 |
| Ethanolamine | 0.019 | 0.012 | 0.016 | 0.008 | 0.017 | 0.009 |
| Formate | 0.055 | 0.036 | 0.050 | 0.011 | 0.064 | 0.036 |
| Fructose | 0.152 | 0.063 | 0.143 | 0.055 | 0.171 | 0.069 |
| Glucose | 3.201 | 1.253 | 3.206 | 0.925 | 3.353 | 1.004 |
| Glutamine | 0.466 | 0.156 | 0.478 | 0.090 | 0.513 | 0.174 |
| Glycolate | 0.034 | 0.012 | 0.031 | 0.009 | 0.033 | 0.012 |
| Histidine | 0.027 | 0.012 | 0.024 | 0.006 | 0.030 | 0.012 |
| Isoleucine | 0.007 | 0.003 | 0.007 | 0.003 | 0.007 | 0.003 |
| Lactate | 2.012 | 0.889 | 1.761 | 0.427 | 2.017 | 0.907 |
| Leucine | 0.014 | 0.007 | 0.016 | 0.006 | 0.017 | 0.008 |
| Lysine | 0.033 | 0.013 | 0.033 | 0.008 | 0.038 | 0.014 |
| Mannose | 0.045 | 0.020 | 0.050 | 0.015 | 0.048 | 0.027 |
| Methionine | 0.014 | 0.010 | 0.015 | 0.005 | 0.016 | 0.010 |
| O-Acetylcarnitine | 0.001 | 0.001 | 0.001 | 0.001 | 0.001 | 0.001 |
| O-Phosphocholine | 0.001 | 0.001 | 0.001 | 0.001 | 0.001 | 0.001 |
| Phenylalanine | 0.015 | 0.008 | 0.015 | 0.004 | 0.017 | 0.008 |
| Pyroglutamate | 0.020 | 0.008 | 0.018 | 0.006 | 0.020 | 0.011 |
| Serine | 0.036 | 0.018 | 0.032 | 0.015 | 0.039 | 0.025 |
| Succinate | 0.002 | 0.001 | 0.002 | 0.001 | 0.002 | 0.001 |
| Threonine | 0.066 | 0.025 | 0.059 | 0.014 | 0.074 | 0.033 |
| Tyrosine | 0.018 | 0.010 | 0.016 | 0.004 | 0.019 | 0.010 |
| Urea | 0.884 | 0.630 | 0.880 | 0.443 | 0.923 | 0.644 |
| Valine | 0.022 | 0.013 | 0.024 | 0.010 | 0.027 | 0.012 |
| myo-Inositol | 0.174 | 0.077 | 0.172 | 0.047 | 0.184 | 0.062 |
| sn-Glycero-3-phosphocholine | 0.002 | 0.001 | 0.002 | 0.001 | 0.002 | 0.001 |

**Suppl. Tab. 1.** Table of CSF annotated metabolite concentrations of CSF aliquots separated into clinical groups.

| **B.I. QUANT-PS**  **Parameter (mmol/L)** | **Mean Value AD**  **(n=26)** | **Standard Deviation AD** | **Mean Value Con**  **(n=29)** | **Standard Deviation Con** | **Mean Value MCI**  **(n=21)** | **Standard Deviation MCI** |
| --- | --- | --- | --- | --- | --- | --- |
| Alanine | 0.43 | 0.08 | 0.50 | 0.11 | 0.48 | 0.08 |
| Creatine | 0.02 | 0.02 | 0.01 | 0.02 | 0.01 | 0.01 |
| Creatinine | 0.08 | 0.01 | 0.08 | 0.02 | 0.09 | 0.02 |
| Glutamic acid | 0.03 | 0.05 | 0.07 | 0.05 | 0.04 | 0.05 |
| Glutamine | 0.73 | 0.11 | 0.73 | 0.07 | 0.75 | 0.08 |
| Glycine | 0.30 | 0.06 | 0.30 | 0.06 | 0.29 | 0.06 |
| Histidine | 0.08 | 0.02 | 0.08 | 0.01 | 0.08 | 0.01 |
| Isoleucine | 0.05 | 0.02 | 0.06 | 0.02 | 0.06 | 0.02 |
| Leucine | 0.09 | 0.03 | 0.10 | 0.02 | 0.11 | 0.04 |
| Lysine | 0.18 | 0.09 | 0.20 | 0.08 | 0.21 | 0.06 |
| Methionine | 0.02 | 0.03 | 0.02 | 0.03 | 0.02 | 0.03 |
| Ornithine | 0.03 | 0.04 | 0.05 | 0.03 | 0.04 | 0.03 |
| Phenylalanine | 0.05 | 0.01 | 0.06 | 0.01 | 0.06 | 0.02 |
| Proline | 0.13 | 0.15 | 0.11 | 0.14 | 0.17 | 0.17 |
| Threonine | 0.08 | 0.07 | 0.11 | 0.08 | 0.10 | 0.07 |
| Tyrosine | 0.06 | 0.02 | 0.07 | 0.01 | 0.06 | 0.02 |
| Valine | 0.22 | 0.05 | 0.24 | 0.05 | 0.23 | 0.06 |
| Acetic acid | 0.01 | 0.01 | 0.02 | 0.03 | 0.01 | 0.01 |
| Citric acid | 0.17 | 0.04 | 0.18 | 0.04 | 0.18 | 0.04 |
| Formic acid | 0.01 | 0.01 | 0.02 | 0.02 | 0.01 | 0.01 |
| Lactic acid | 2.09 | 0.61 | 2.60 | 0.92 | 2.26 | 0.65 |
| 3-Hydroxybutyric acid | 0.06 | 0.07 | 0.08 | 0.10 | 0.06 | 0.08 |
| Acetoacetic acid | 0.01 | 0.03 | 0.02 | 0.02 | 0.01 | 0.02 |
| Acetone | 0.02 | 0.01 | 0.03 | 0.02 | 0.02 | 0.01 |
| Pyruvic acid | 0.07 | 0.03 | 0.07 | 0.04 | 0.09 | 0.04 |
| Glucose | 5.27 | 0.97 | 5.58 | 1.10 | 6.18 | 1.33 |
| Dimethylsulfone | 0.02 | 0.07 | 0.01 | 0.01 | 0.01 | 0.01 |
| Fischer’s ratio (calculated) | 3.22 | 0.68 | 3.19 | 0.62 | 3.45 | 0.85 |
| Gln/Glc (calculated) | 0.14 | 0.03 | 0.14 | 0.02 | 0.13 | 0.03 |
| Lac/Glc (calculated) | 0.42 | 0.18 | 0.47 | 0.13 | 0.38 | 0.12 |

**Suppl. Tab. 2.** Table of blood serum annotated metabolite concentrations of serum aliquots separated into clinical groups.

| **Compound (CSF)** | **F value** | **P value** | **-log10(p)** | **FDR** |
| --- | --- | --- | --- | --- |
| Protein background***  ↑AD, MCI | 9.5497 | 0.0002 | 3.6552 | 0.0100 |
| 2-Hydroxybutyrate*  ↓AD, MCI | 4.4231 | 0.0156 | 1.8059 | 0.3382 |
| Alpha-ketoisovaleric acid*  ↑AD, MCI | 3.5822 | 0.0332 | 1.4791 | 0.3382 |
| Ascorbate*  ↑AD, MCI | 3.4270 | 0.0382 | 1.4180 | 0.3382 |
| 3-Hydroxyisobutyrate*  ↓AD, MCI | 3.2110 | 0.0465 | 1.3325 | 0.3382 |
| Acetoacetate*  ↓AD, MCI | 3.1846 | 0.0476 | 1.3221 | 0.3382 |

**Suppl. Tab. 3.** ANOVA results of CSF metabolites significantly (by p value) comparing three patient groups with Alzheimer’s disease (AD; n = 29), healthy controls (Con; n = 20), and patients with mild cognitive impairment (MCI; n = 22) in the cerebrospinal fluid analytes. Statistical significance: * – P ≤ 0.05, ** – P ≤ 0.01, *** – P ≤ 0.001.

| **CSF compounds and metadata** | **AD vs Con** | **P value** | **MCI vs Con** | **P value** | **AD vs MCI** | **P value** |
| --- | --- | --- | --- | --- | --- | --- |
| Protein background | ** [***]  ↑AD | 0.0014  [0.0005] | ***  ↑MCI | 0.000123  [0.000007] | [*  ↓AD] | [0.0379] |
| 2-Hydroxybutyrate | **  ↓AD | 0.0057  [0.0074] | *  ↓MCI | 0.047072  [0.039072] |  |  |
| Histidine |  |  | * [ns]  ↑MCI | 0.011 |  |  |
| Alpha-ketoisovaleric acid | *  ↑AD | 0.0185  [0.0256] |  |  |  |  |
| Ascorbate |  |  | *[**]  ↑MCI | 0.0185  [0.0079] |  |  |
| 3-Hydroxy-isobutyrate | *  ↓AD | 0.0246  [0.0198] | [*  ↓MCI] | [0.0207] |  |  |
| 2-Hydroxy-isovalerate | *  ↑AD | 0.04435  [0.0419] |  |  | *  ↑AD | 0.0225  [0.0409] |
| Acetoacetate | * [ns]  ↓AD | 0.03402 | *  ↓MCI | 0.035  [0.0478] |  |  |
| Mannose | * [ns]  ↓AD | 0.04090 |  |  |  |  |
| Valine |  |  |  |  | *  ↓AD | 0.0417  [0.0438] |
| Creatine |  |  |  |  | * [ns]  ↑AD | 0.0489 |
| Fructose |  |  | [**  ↑MCI] | [0.00621] |  |  |
| Formate |  |  |  |  | [*  ↓AD] | [0.0360] |
| CSF p-Tau | [***  ↑AD] | [8.41E-14] | [***  ↑MCI] | [0.000176] | [***  ↑AD] | [0.000116] |
| CSF h-Tau | [***  ↑AD] | [2.90E-13] | [***  ↑MCI] | [0.000015] | [***  ↑AD] | [0.000109] |
| AmPos | [***  ↑AD] | [4.49E-9] | [***  ↑MCI] | [0.000009] |  |  |
| CSF Abeta1-42 | [***  ↓AD] | [1.15E-6] | [**  ↓MCI] | [0.009319] |  |  |
| MMSE |  |  |  |  | [***  ↓AD] | [0.000647] |

**Suppl. Tab. 4.** T-test results of CSF metabolites (by p value) comparing two patient groups (pairwise between Alzheimer’s disease (AD; n = 29), healthy controls (Con; n = 20), and mild cognitive impairment group (MCI; n = 22) in the cerebrospinal fluid analytes; t-test statistical analysis results (shown in square brackets) from data entries that included available clinical metadata in a pairwise comparison between Alzheimer’s disease (AD; n = 25), healthy controls (Con; n = 15), and mild cognitive impairment group (MCI; n = 18). Statistical significance: * – P ≤ 0.05, ** – P ≤ 0.01, *** – P ≤ 0.001, ns – not significant. Used abbreviations: AmPos – amyloid positive status, MMSE – mini-mental state examination.

| **Compound (Serum)** | **F value** | **P value** | **-log10(p)** | **FDR** |
| --- | --- | --- | --- | --- |
| Glutamic acid**  ↓AD, MCI | 4.961 | 0.010 | 2.020 | 0.286 |
| Ornithine*  ↓AD, MCI | 3.559 | 0.033 | 1.475 | 0.311 |
| Glucose*  ↑ MCI | 3.402 | 0.039 | 1.413 | 0.311 |
| Acetoacetic acid*  ↓AD, ↓↓MCI | 3.327 | 0.041 | 1.383 | 0.311 |

**Suppl. Tab. 5.** ANOVA results of serum metabolites significantly (by p value) comparing three patient groups with Alzheimer’s disease (AD; n = 26), healthy controls (Con; n = 29), and patients with mild cognitive impairment (MCI; n = 21) in the blood serum analytes. Statistical significance: * – P ≤ 0.05, ** – P ≤ 0.01.

| **Compound/ lipoprotein entry (Serum)** | **AD vs Con** | **P value** | **MCI vs Con** | **P value** | **AD vs MCI** | **P value** |
| --- | --- | --- | --- | --- | --- | --- |
| Glutamic acid | **  ↓AD | 0.00253 |  |  |  |  |
| Ornithine | *  ↓AD | 0.01143 |  |  |  |  |
| Acetoacetic acid |  |  | *  ↓MCI | 0.0117 |  |  |
| Glucose |  |  | *  ↑MCI | 0.0217 | *  ↓AD | 0.0386 |
| Lactate/glucose (Lac/Glc) |  |  | *  ↓MCI | 0.0292 |  |  |
| Glutamine/glucose (Gln/Glc) |  |  |  |  | *  ↑AD | 0.0348 |
| MMSE | ***  ↓AD | 1.26E-14 | ***  ↓MCI | 0.0000016 | ***  ↓AD | 0.0000015 |
| CSF h-Tau |  |  |  |  | *  ↑AD | 0.0264 |
| Ch-est inh  (cholinesterase inhibitor medication status) | ***  ↑AD | 0.0000096 | ***  ↑MCI | 0.0005045 |  |  |
| Age | ***  ↓AD | 0.0006524 |  |  |  |  |
| ApoE4 | *  ↑AD | 0.0232680 | *  ↑MCI | 0.0366420 |  |  |

**Suppl. Tab. 6.** T-test results of serum metabolites and available clinical metadata (Abeta, tau – only for AD and MCI groups) data significantly (by p value) comparing two patient groups (pairwise between Alzheimer’s disease (AD; n = 26), healthy controls (Con; n = 29), and mild cognitive impairment group (MCI; n = 21) in the blood serum analytes. Serum metabolites and available clinical metadata significances were tested separately. Statistical significance: * – P ≤ 0.05, ** – P ≤ 0.01, *** – P ≤ 0.001. Used abbreviations: MMSE – mini-mental state examination, Ch-est inh – cholinesterase inhibitor.

| Compounds and metadata: | **FC value** | **log_2_(FC)** | **FDR** |
| --- | --- | --- | --- |
| Creatine (serum)* | 4.716 | 2.238 | 0.001 |
| AD-M < AD-F |  |  |  |
|  |  |  |  |
| Glycine (serum)* | 1.274 | 0.349 | 0.075 |
| AD-M < AD-F |  |  |  |
| Creatinine (serum)** | 0.753 | -0.409 | 0.004 |
| Con-M > Con-F |  |  |  |
|  |  |  |  |
| Creatine (serum)* | 4.54 | 2.183 | 0.01 |
| Con-M < Con-F |  |  |  |
|  |  |  |  |
| Formic acid (serum)* | 3.152 | 1.656 | 0.01 |
| Con-M < Con-F |  |  |  |

Suppl. Tab. 7. Notable results of whole-cohort serum metabolites and sex-based entries significantly (by p value via a Volcano analysis) comparing two patient groups. Statistical significance: * p ≤ 0.05, ** p ≤ 0.01. FC – fold change. M – male. F – female.

| Compounds and metadata: | **F value** | **p value** | **FDR** |
| --- | --- | --- | --- |
| 2-Hydroxybutyrate (CSF) | 7.489 | 0.002 | 0.079 |
| Con-M > AD-M or MCI-M |  |  |  |
|  |  |  |  |
| Acetoacetate (CSF) | 5.92 | 0.006 | 0.087 |
| Con-M > AD-M or MCI-M |  |  |  |
|  |  |  |  |
| Protein background (CSF) | 5.77 | 0.006 | 0.087 |
| Con-M < AD-M or MCI-M |  |  |  |
|  |  |  |  |
| 1,5-Anhydrosorbitol (CSF) | 5.538 | 0.008 | 0.087 |
| Con-M < AD-M or MCI-M |  |  |  |

**Suppl. Tab. 8**. Notable results of whole-cohort CSF sex-based entries significantly (by p value via ANOVA) comparing 3 patient groups. M – male. F – female.

| Compounds and metadata: | **r value** | **p value** | **FDR** |
| --- | --- | --- | --- |
| Glucose (CSF) ▪ Age | -0.315 | 0.008 | 0.177 |
| Lactate (CSF)* ▪ Sex | 0.362 | 0.002 | 0.03 |
| Creatine (CSF) * ▪ Sex | 0.358 | 0.002 | 0.03 |
| Valine (CSF) ▪ Sex | -0.328 | 0.005 | 0.06 |
| Creatine (serum)*** ▪ Sex | 0.531 | 8E-07 | 1.32E-05 |
| Glycine (serum)** ▪ Sex | 0.455 | 0.00004 | 0.00038 |
| Leucine (serum)* ▪ Sex | -0.319 | 0.0050 | 0.0402 |

**Suppl. Tab. 9**. Notable results of whole-cohort CSF and serum metabolites and age/sex-based entries significantly (by p value via PatternHunter, Pearson correlations |r| > 0.30) comparing two patient groups. Statistical significance: * p ≤ 0.05, ** p ≤ 0.01, *** p ≤ 0.001.

| Compound ratios: | **AUC value** | **p value** |
| --- | --- | --- |
| Valine S/Lysine C | 0.76471 | 0.0047069 |
| Creatinine C/Lysine C | 0.74332 | 0.0060719 |
| Isoleucine C/Valine C | 0.74332 | 0.0114150 |
| Creatinine C/Glutamine C | 0.73262 | 0.0106680 |
| **Valine S/Valine C** | **0.72460** | **0.0110990** |
| Creatine C/Glutamine C | 0.72460 | 0.0192490 |
| Creatinine C/Histidine C | 0.72460 | 0.0169210 |
| Creatine C/Lysine C | 0.71658 | 0.0070082 |
| Tyrosine S/Lysine C | 0.71390 | 0.0220280 |
| Creatinine C/Glucose C | 0.71390 | 0.0211250 |

**Suppl. Tab. 10**. ROC analysis results of CSF (C) together with serum (S) metabolites and metadata entries significantly (by p value): comparing two patient groups (AD; n = 22) and (MCI; n = 17) in the CSF and blood serum analytes. AUC – area-under-curve.
